# Characterisation of post-translational and transcriptional reprogramming of the immune response to ISAV and IPNV infections in salmon head kidney cells

**DOI:** 10.3389/fimmu.2025.1532917

**Published:** 2025-10-17

**Authors:** Robert Stewart, Xoel Souto Guitián, Ophélie Gervais, Yehwa Jin, Sarah J. Salisbury, Maeve Ballantyne, Samuel A. M. Martin, Beatriz Orosa-Puente, Diego Robledo

**Affiliations:** ^1^ The Roslin Institute and Royal (Dick) School of Veterinary Studies, The University of Edinburgh, Edinburgh, United Kingdom; ^2^ University of Santiago de Compostela, Santiago de Compostela, Spain; ^3^ Centre Scientifique de Monaco, Monaco, Monaco; ^4^ The Center for Aquaculture Technologies, San Diego, CA, United States; ^5^ Scottish Fish Immunology Research Centre, School of Biological Sciences, University of Aberdeen, Aberdeen, United Kingdom

**Keywords:** aquaculture, immune response, ubiquitin, virus, proteomics, RNA sequencing

## Abstract

Viral diseases remain a major barrier to the sustainable production of farmed fish, primarily attributable to the absence of effective prevention and treatment options. Understanding host-pathogen interactions can guide the development of vaccines, antiviral therapies, or gene editing strategies. Ubiquitination is a post-translational modification capable of regulating protein activation, structure, and degradation. As such, it is known to regulate many aspects of immune functions in model species, but is currently understudied in fish. This study leverages ubiquitin-enriched mass spectroscopy complemented with RNA sequencing to characterise the role of ubiquitination in response to infection. A challenge experiment was conducted by infecting Atlantic salmon head kidney (SHK-1) cells with Infectious salmon anaemia virus (ISAV) and Infectious pancreatic necrosis virus (IPNV). At 24 and 48 hours post-infection, dramatic changes were observed in the global ubiquitination state of host proteins. Many post-translational modifying proteins increased in abundance upon ISAV infection, whilst IPNV infection resulted in a reduction in abundance of many of these proteins. Transcriptomics showed a delay in the activation of the antiviral response to ISAV infection, with major upregulation of genes associated with immune pathways only at 48h. On the contrary, IPNV infection resulted in upregulation of classic innate immune response genes at both timepoints. Clear activation of Rig-like receptor pathways is demonstrated in both infections, in addition to upregulation of both conserved and novel antiviral TRIM E3 ubiquitin ligase genes. Network analysis identified clusters of immune genes and putatively regulatory proteins showing differential ubiquitination upon viral infection. This study highlights the capacity of post-translational control of the host innate immune response to viruses in Atlantic salmon. Clear differences in ubiquitination between the two viruses indicate either virus-specific post-translational regulation or viral antagonism of the immune response. Additionally, the ubiquitination of various proteins was linked to the regulation of innate immune pathways, suggesting a direct role of ubiquitination in the regulation of antiviral responses.

## Introduction

### Aquaculture and disease

Aquaculture is a crucial tool for meeting the increasing demand for healthy protein - the UN predicts that food demand will double by 2050 as the global population reaches almost 10 billion (1–3). In 2020, 17% of global animal-source protein consumed was derived from combined aquaculture and fisheries, however, any opportunities to sustainably increase wild fish harvest are limited (4, 5). Accordingly it is predicted that the aquaculture sector will grow by up to 74% over the next 25 years to reflect the ever-increasing demand for protein (5). While aquaculture food production has multiple advantages over conventional, land-based animal protein production systems including better food conversion efficiency (6), low carbon footprint (7) and lower competitive land use (8), the growth of the sector is limited by infectious diseases - one of the leading causes of production losses in fish farming (9) – in 2024 32.9% of farmed salmon mortalities were attributed to infectious disease in Norway (10).

### Viral diseases in salmon aquaculture

Despite capacity to cause high levels of mortality in marine-raised Atlantic salmon (11), there are few effective prophylactic and therapeutic options for viral diseases affecting salmonids (12–14). In particular, Infectious Salmon Anaemia Virus (ISAV) and Infectious Pancreatic Necrosis Virus (IPNV) can have catastrophic impacts on the aquaculture sector. ISAV was first reported in Norway in 1984, and has two distinct phenotypes; the low pathogenic HPR0 strain and the highly pathogenic HPRΔ strain. Mortality rates due to ISAV are highly variable depending on virus strain, host genetics and infection conditions, yet mortalities can reach over 90% if no preventative or therapeutic measures are implemented (15). IPNV was first described in 1941, although it was initially named ‘whirling sickness’ (16), before being renamed in 1951. Unlike ISAV, which typically affects the marine phase, IPNV predominantly affects young fry, where susceptible fish at certain ages can suffer close to 100% moralities (17). Despite the welfare, economic, and environmental impact of these viruses, both have limited prevention and treatment options. Atlantic salmon are commonly vaccinated against ISAV in many production countries, however, current vaccines do not yet offer complete protection (18). Additionally, there is currently no vaccine available for IPNV (19, 20), for which genomic techniques have facilitated partially protective selective breeding for IPNV resistance (21). Further insights into the host-pathogen interaction of these viruses have the potential to aid in the development of more effective vaccines and help identify targets for selective breeding and gene editing to improve host disease resistance.

### Ubiquitin in the immune response

Post-translational modification pathways are key regulators of both the innate and adaptive immune response (22, 23). In particular, ubiquitination – the addition to a protein of ubiquitin, a small 76 amino acid peptide highly conserved across eukaryotes (24) - has a broad range of functional effects on the substrate protein, including changes in activity, stability, compartmentalisation, and conformation. This dynamic and complex modification can lead to the activation of immune pathways; as seen by the ubiquitin-dependant activation of RIG-I upon sensing of dsRNA to trigger IFN signalling (25), deactivation by targeting regulatory proteins for proteasomal degradation (26), or direct antiviral mechanisms such as the blocking of viral entry by the Ubiquitin ligase TRIM5 (27). Due to the co-evolution of host and virus, ubiquitin and the ubiquitin proteasome system (UPS) can have both antiviral and pro-viral roles; as seen in Dengue virus infection where ubiquitination is harnessed for viral uncoating (28), and SARS-CoV-2 has been shown to hijack its hosts UPS to inhibit STAT2-mediated interferon response (29).

### Summary and aim

Immune regulation by ubiquitination and ubiquitin-like modifications is crucial for a balanced response to pathogens. In model species, the role of ubiquitination has been demonstrated in both the activation and deactivation of both the innate and adaptive immune response (30). Teleost fish have a greater reliance on the innate immune response than mammals (31), hence post-translational modifications may also have a more pivotal role in host-pathogen interactions in fish. Therefore, this research aims to characterise the global changes in ubiquitome in response to viral infections in Atlantic salmon, in particular to the changing ubiquitination state of host immune signalling pathways, and to determine the impact that this has on the transcriptome. This research has focused on understanding the role of ubiquitination in response to viral infections (ISAV and IPNV) in Atlantic salmon head kidney cells. ISAV and IPNV were studied due to their impact on fresh water and sea water production of Atlantic salmon, in addition to emerging evidence of the post-translational nature of the immune response to both of these viruses (21, 32). Our results reveal clear virus-specific changes in ubiquitination patterns in a salmonid cell line, which can be connected to changes in immune-related pathways at both the proteomic and transcriptomic levels.

## Results

### Global ubiquitination changes in response to viral infection

**Figure 1:**
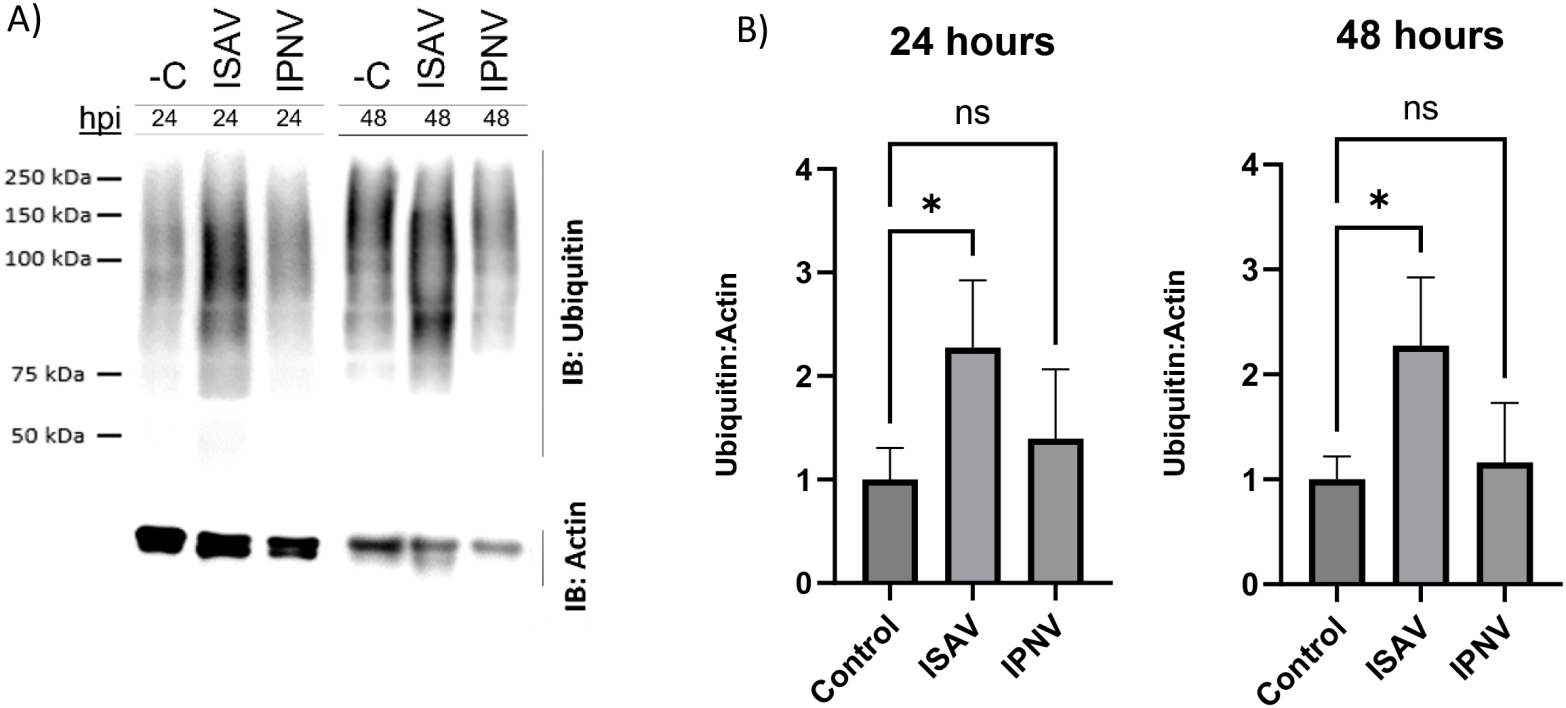
Global ubiquitination state of infected cells. **(A)** Representative blots of Anti-Ubiquitin western blots from ISAV and IPNV infected whole cell lysate at 24 hpi and 48 hpi. C- denotes negative control (uninfected, time-matched samples). IB: refers to antibodies used for an immune-blot. 24 and 48 hour timepoints were run on separate gels, and a composite image was created. Poncea total protein stain is included in **Supplementary Figures 1A, B**. **(B)** Quantification and analysis of Western blot bands performed in Licor Image Studio. Fold changes are relative to time-matched control. Errors bars represent standard deviation. One way ANOVA was performed to check for statistical significance, where (*) represents p value <0.05 with a Dunnett’s multiple comparison test.

An Atlantic salmon head kidney cell line (SHK-1) was infected with ISAV and IPNV, and cell lysates were collected at early innate immune response timepoints—24 and 48 hours post-infection. To assess the global host ubiquitin response to each viral infection, western blot analysis was conducted on whole-cell lysates from ISAV- and IPNV-infected cultures at these timepoints (**Figure 1A**). Pan-linkage-specific ubiquitin blotting revealed a significant increase in total protein ubiquitination following ISAV infection compared to uninfected, time-matched controls. In contrast, IPNV infection did not lead to a significant increase in overall protein ubiquitination at either 24 or 48 hours post-infection (**Figure 1B**).

### Viral kinetics and infection metrics

**Figure 2:**
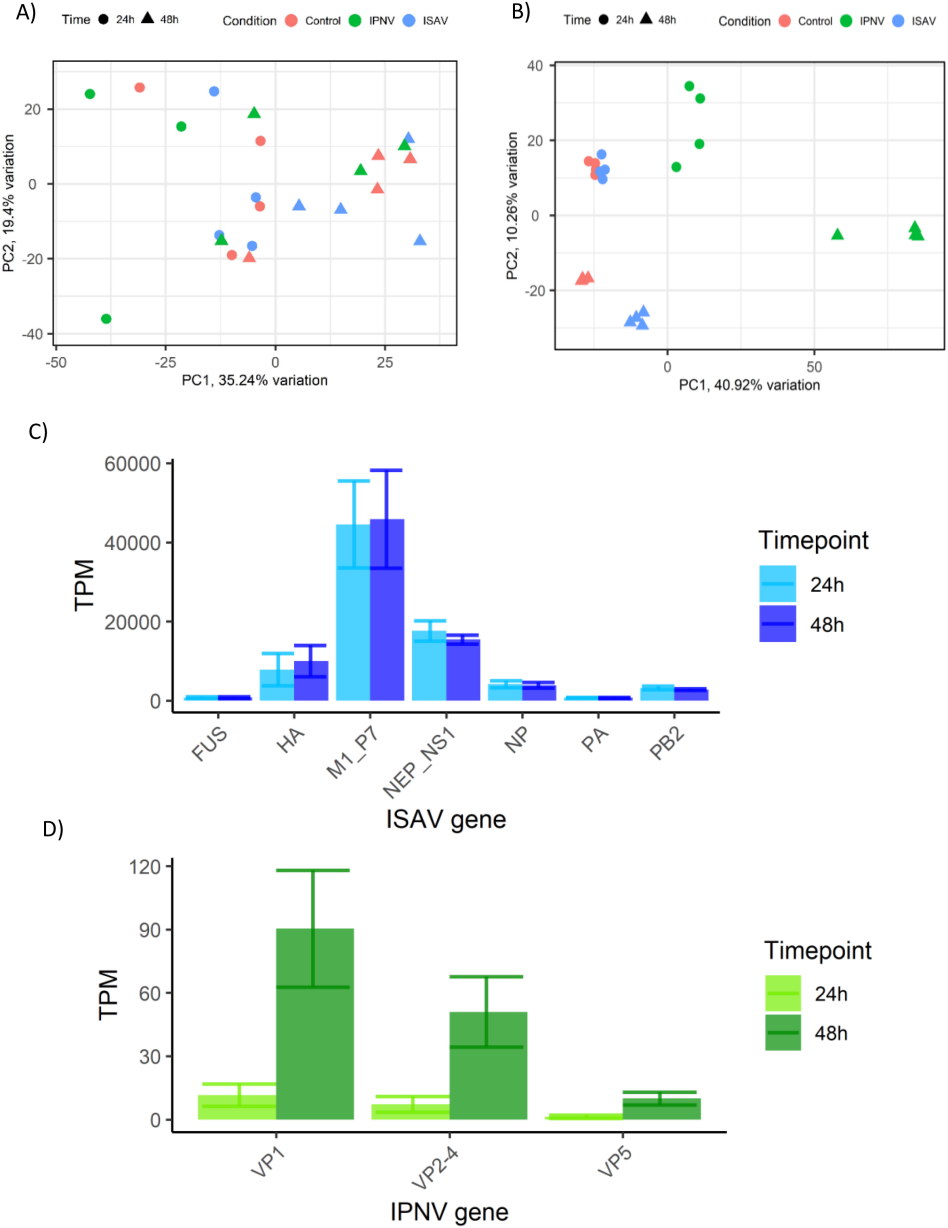
Infection metrics of ISAV and IPNV infection of SHK-1 cells. **(A)** Principle component analysis of 1418 quantified ubiquitin-enriched proteins detected by label-free mass spectroscopy. **(B)** Principal component analysis of host transcripts in response to ISAV and IPNV infection, cut-off values p value <10e-6, <0.5 Log2FC. proteomics data (top left) and RNA seq (top right). **(C)** Quantification of ISAV viral genes/segments in the RNA sequencing data, measured as Transcripts Per Million (TPM). **(D)** Quantification of IPNV viral genes/segments in the RNA sequencing data, measured as Transcripts Per Million (TPM).

Ubiquitin-enrichment proteomics was utilised to characterise the post-translational response observed upon infection. Infected and control whole cell lysates were enriched for ubiquitin proteins using Ubiquilin pull-down, and quantified via mass spectroscopy. A total of 3224 proteins were identified using label-free mass spectroscopy (all detectable proteins), which was filtered to 1418 proteins detected in all samples and identified against the current Uniprot proteome for Atlantic Salmon (UP000087266). Principal component analysis of 1418 proteins shows a lack of clustering by infection, with samples primarily split by time along PC1 (**Figure 2A**).

RNA sequencing of infected and control samples revealed the transcriptomic response occurring concurrently with changes in the host protein ubiquitination state. Principal component analysis of RNA sequencing data showed distinct clustering of experimental groups, with the exception of Control 24-hour and ISAV 24-hour sample groups (**Figure 2B**), which clustered together.

Viral transcripts for ISAV and IPNV were quantified from RNA sequencing data to identify viral kinetics in infected cells (**Figures 2C, D**). The expression level of ISAV was much higher than that of IPNV, consistent with the higher multiplicity of infection (MOI). However, while IPNV levels increased ~4 fold between 24 and 48h, ISAV levels remained unchanged.

### Transcriptional response follows post-translational response to ISAV infection

**Figure 3:**
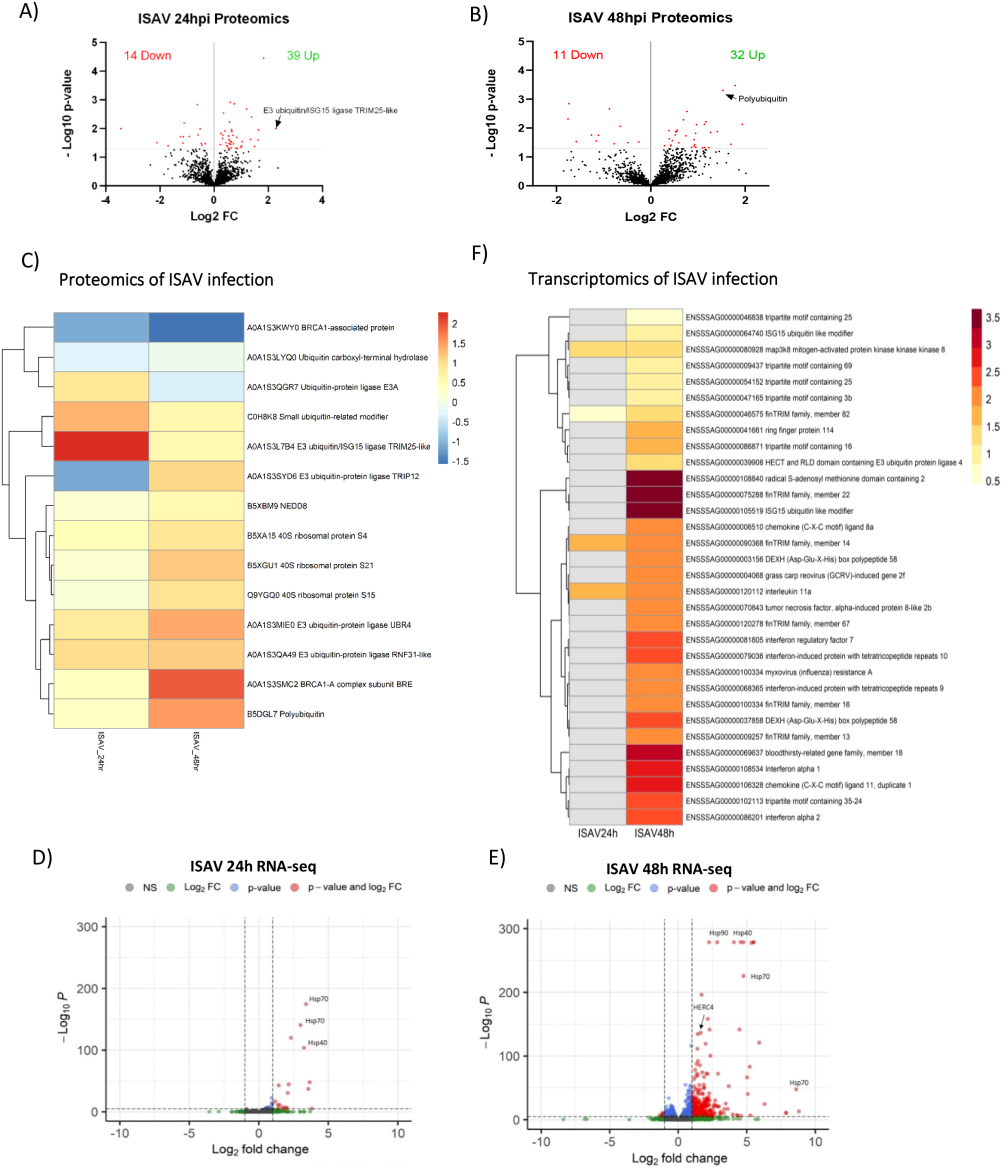
Proteomics and transcriptomics of ISAV infection. A&B) Volcano plots of host proteins upon ISAV infection of SHK-1 cells after 24 **(A)** or 48 hours **(B)**, significance threshold (padj<0.05) indicated by dotted line. The total number of significant proteins increased/decreased upon infection is displayed. **(C)** Heat map of significantly different abundant proteins upon infection. Colours represent Log2 Fold change. Heatmap of individual samples is shown in **Supplementary Figures 2D, E**) Volcano plots of transcripts upon ISAV infection after 24 **(D)** or 48 hours **(E)**, significance threshold (padj <0.05, Log2FC >1) indicated by dotted line. **(F)** Heat map of significantly differently expressed genes relating to infection and ubiquitination. Colours represent Log2 Fold change. Non-significant values are displayed in grey.

Western blot analysis of the host proteome following ISAV infection revealed significant changes in protein ubiquitination, leading to an increase in the abundance of ubiquitinated and ubiquitin-associated proteins. Proteomics analysis reflects the changes seen in western blot, with 74% of differentially abundant proteins increasing in abundance for both 24- and 48-hour time points (**Figures 3A, B**).

Mass spectrometry detected 17 differentially ubiquitinated proteins at 24 hours (10 with increased ubiquitination and 7 decreased) and 17 at 48 hours (11 increased and 6 decreased) (**Supplementary Files 1**, **3**, **Figure 3**), with only 2 proteins overlapping between timepoints. Many of these were post-translational regulatory-related proteins (**Figure 3C**). Ubiquitination pathway proteins that increase in abundance upon ISAV infection include E3 ligases; UBR4 (2.7 fold change), RNF31-like protein (2.2 fold change), Ubiquitin/ISG15 ligase TRIM25-like (4.9 fold change), – in addition to polyubiquitin (2.9 fold change), BRCA1-A complex subunit (3.8 fold change), and small ubiquitin-related modifier (2.5 fold change). Multiple components of the ribosome increase in abundance, including subunits S21, S15 and S4. Post-translational proteins reducing in abundance include BRCA1-associated proteins (-0.3 fold change) and Ubiquitin carboxyl-terminal hydrolase (-0.8 fold change). Notably, two ubiquitin-ligase proteins are increased in abundance at one timepoint, and decreased at another; Ubiquitin protein ligase E3A (1.8 fold change 24hrs, -0.8 fold change 48hrs) and E3 Ubiquitin-protein ligase TRIP12 (-0.5 fold change 24hrs, 2 fold change 48hrs).

Principal component analysis of infected SHK-1 cells demonstrated a mild transcriptomic response to ISAV infection at 24 hours (**Figure 2A**), contrary to the strong post-translational response previously described. Indeed, ISAV infection resulted in a limited transcriptomic response at 24 hours, with only 36 genes significantly upregulated (p adj <0.05, FC >2; **Figure 3D**, **Supplementary File 2**). Upregulated genes of note include map3k8, a regulator of the TNFα pathway; interleukin 11, a modulator of inflammation and immunity; and FinTRIM family member 14, a member of the expanded subfamily of TRIM proteins in fish. GO term analysis of transcriptomics of ISAV infection at 24 hours produced limited relevant enriched GO terms and as such data is not shown.

In contrast, an important transcriptomic response was observed 48 hours post-ISAV infection, with 647 upregulated genes (and 45 down-regulated) (**Figure 3E**, **Supplementary File 2**). Amongst the upregulated genes are classical markers of innate interferon antiviral response, best exemplified by RSAD2 (5.7 fold change), interferon alpha 1 (6.4 fold change), RIG-I (ddx58) (3.1 fold change), LGP2 (dhx58) (4.8 fold change), IFIT8 (3.6 fold change), IFIT9 (4.4 fold change), IFIT10 (5.0 fold change), IRF7 (5.3 fold change), and Mx2 (4.4 fold change) (**Figure 3F**). Many members of the conserved (TRIM) and novel (FinTRIM and Blood Thirsty) TRIM gene family are significantly upregulated in response to infection; 5 types of TRIM genes (*trim35, trim25, trim3b, trim69* and *trim16*), 8 different FinTRIM genes (*finTRIM72, finTRIM12, finTRIM66, finTRIM82, finTRIM67, finTRIM13, finTRIM14* and *finTRIM16*) and two blood thirsty gene family members (*bty18, bty4*) are upregulated. Two *Trim25* genes were upregulated in response to ISAV infection, mapped to chromosomes 2 (ENSSSAG00000054152) and 12 (ENSSSAG00000046838), reflecting the paralogues arising from salmonid whole genome duplication (33); the *Trim25* paralogue located on chromosome 12 is upregulated only 1.4-fold (padj = 0.004882), whilst *trim25* paralogue on chromosome 2 is upregulated with a greater magnitude (2.1-fold) and significance (padj = 1.80E-11).

**Figure 4:**
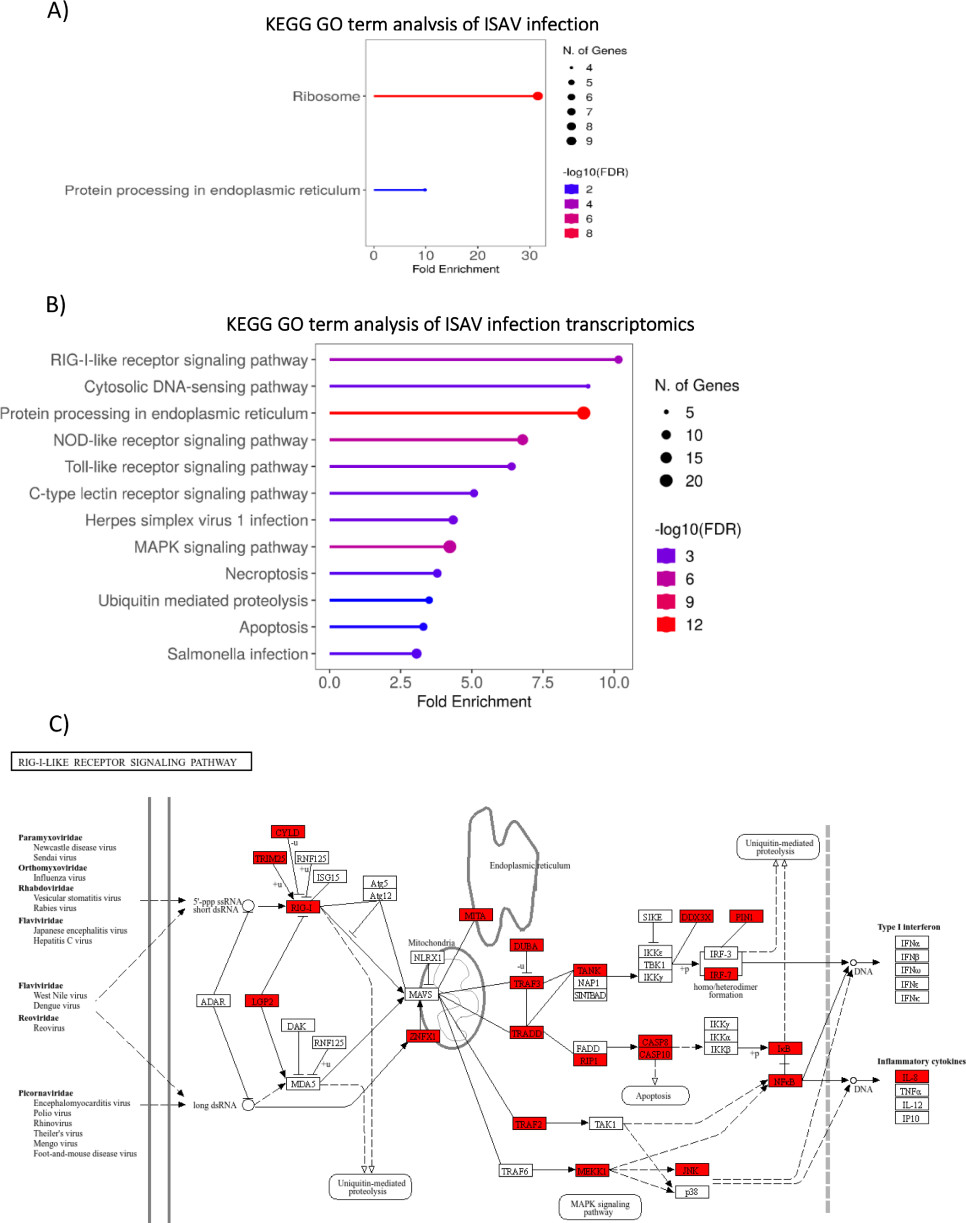
GO and KEGG analysis of ISAV infection – **(A)** GO Term enrichment analysis of ISAV infection, 48 hours post infection **(B)** Enriched GO term analysis of significantly upregulated transcripts (padj value <0.05, FC >2) from ISAV infection at 48 hours post infection. GO Term analysis was performed on closest homologs of salmon genes in model species; Zebrafish. **(C)** KEGG pathway annotation of zebrafish genes homologous to Atlantic Salmon genes upregulated in the RIG-LIKE receptor pathway in response to ISAV Infection. Upregulated genes are highlighted in red. GO term analysis and KEGG pathway annotation was performed with R shiny GO (66).

GO term enrichment analysis of differently abundant proteins for ISAV infection was limited (**Figure 4A**), but revealed significant enrichment ribosomal pathways (>30 fold enrichment) and protein processing in the endoplasmic reticulum (>10 fold enrichment). GO term analysis of upregulated genes (**Figure 4B**) demonstrates clear activation of the innate immune pathogen recognition receptor pathways (PRR), including RIG-I-like receptor (RLR) signalling, Cytosolic DNA sensing, NOD-like receptor signalling (NLR), Toll-like receptor signalling (TLR) and C type lectin receptor signalling (CTLR). Multiple components of the protein production pathways are enriched including protein processing in the endoplasmic reticulum and ubiquitin-mediated proteolysis, the latter GO term being consistent with the global upregulation of ubiquitin signalling observed in the western blot. Further, upregulation of the RLR pathway is demonstrated in **Figure 4C**, where upregulated genes from ISAV-infected SHK-1 cells at 48 hours post-infection are able to almost recapitulate the whole RLR pathway.

### IPNV infection induces a rapid transcriptomic response but limited post-translational response

**Figure 5:**
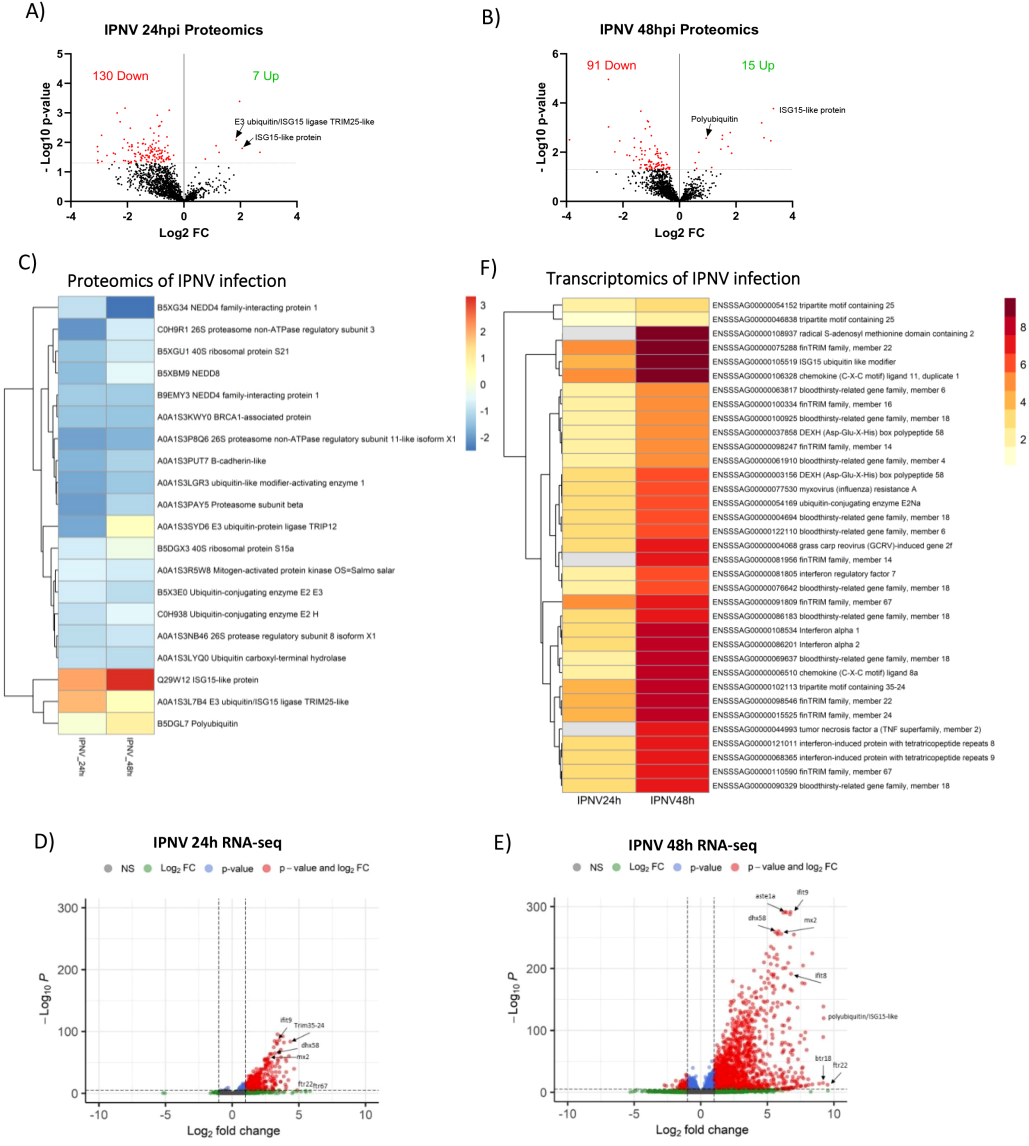
Proteomics and transcriptomics of IPNV infection. **(A, B)** Volcano plots of host proteins upon IPNV infection of SHK-1 cells after 24 **(A)** or 48 hours **(B)**, significance threshold (padj<0.05) indicated by dotted line. The total number of significant proteins increased/decreased upon infection is displayed. **(C)** Heat map of significantly different abundant proteins upon infection. Colours represent Log2 Fold change. Heatmap of individual samples is shown in **Supplementary Figures 2D, E** Volcano plots of transcripts upon IPNV infection after 24 **(D)** or 48 hours **(E)**, significance threshold (padj <0.05, Log2FC >1) indicated by dotted line. **(F)** Heat map of significantly differently expressed genes relating to infection and ubiquitination. Colours represent Log2 Fold change. Non-significant values are displayed in grey.

Western blot analysis of IPNV-infected cells indicated limited changes in the ubiquitin state of host proteins at 24 and 48 hours post infection. Further proteomics analysis following enrichment for ubiquitinated and ubiquitin-associated proteins revealed a reduction in the abundance of ubiquitinated/ubiquitin-associated proteins after infection, with 95% and 86% of significantly differently abundant proteins being reduced at 24 and 48 hours, respectively (**Figures 5A, B**). Only 6 ubiquitinated proteins showed increased abundance at 24h, and 11 at 48h, but 4 of them were common (**Supplementary File 3**, **Figure 3**). Intriguingly, amongst the very few proteins increasing in abundance after IPNV infection are ISG-15-like protein (both at 24 and 48 hours, 4 and 10 fold change, respectively), Polyubiquitin (1.9 fold change) and TRIM25-like E3 ubiquitin/ISG15 ligase (3.6 fold change) (**Figure 5C**).

The majority of differentially ubiquitinated proteins upon IPNV infection are reduced, with 86 showing reduced levels at 24h (and 6 increased) and 46 at 48h (and 11 increased) (**Supplementary File 1**). Most of them are time-specific, with just 11 shared between timepoints (**Supplementary File 3**). Amongst the proteins showing reduced levels are ubiquitin-like modifier-activating enzyme 1 (-0.3 fold change), Ubiquitin-conjugating enzyme E2 E3 (-0.5 fold change), Ubiquitin carboxyl-terminal hydrolase (-0.5 fold change), Nedd4 family interacting protein (-0.2 fold change), proteasome subunits (-0.3 fold change), NEDD8 (-0.4 Fold change), and multiple ribosomal proteins (-0.4 fold change) (**Figure 5C**).

IPNV infection induced a rapid transcriptomic response with 435 upregulated genes at 24 hours (and only 7 downregulated), and 2418 at 48 hours post-infection (463 downregulated) (**Figures 5D, E**, **Supplementary File 2**). At 24 hours, classic markers of innate antiviral response are upregulated, including but not limited to rsad2, IFIT8, IFIT9, IFIT10, IFN, irf3 and multiple components on the Rig-like receptor signalling pathway; DEXH box peptide (LGP2), STAT1a and Mx genes. Two members of the TRIM protein family (*trim25* and *trim107)*, nine members of the expanded FinTRIM family (*finTRIM7*, f*inTRIM12*, f*inTRIM13*, f*inTRIM14*, f*inTRIM16*, f*inTRIM22*, f*inTRIM24*, f*inTRIM66* and f*inTRIM67*), and four members of the bloodthirsty TRIM-like genes (bty4, bty6, bty18 and bty26) are differentially upregulated. Polyubiquitin/ISG15 like protein (a ubiquitin-like modifier) is highly upregulated, as is ubiquitin conjugating enzyme E2Na and HECT and RLD domain containing E3 ligases 3 and 4. Most of these genes are also upregulated at 48 hours post-infection, and an additional 2418 genes were upregulated, including finTRIM82 and tumour necrosis factor alpha (TNFα) (**Figure 5F**). IPNV infection at 48 hours induced a higher upregulation of chromosome 2 copy of *trim25* (10.7 fold change), relative to the chromosome 12 copy (4.9 fold change), reflecting the orthologue-specific observations of the host response to ISAV infection, but with much higher magnitude.

**Figure 6:**
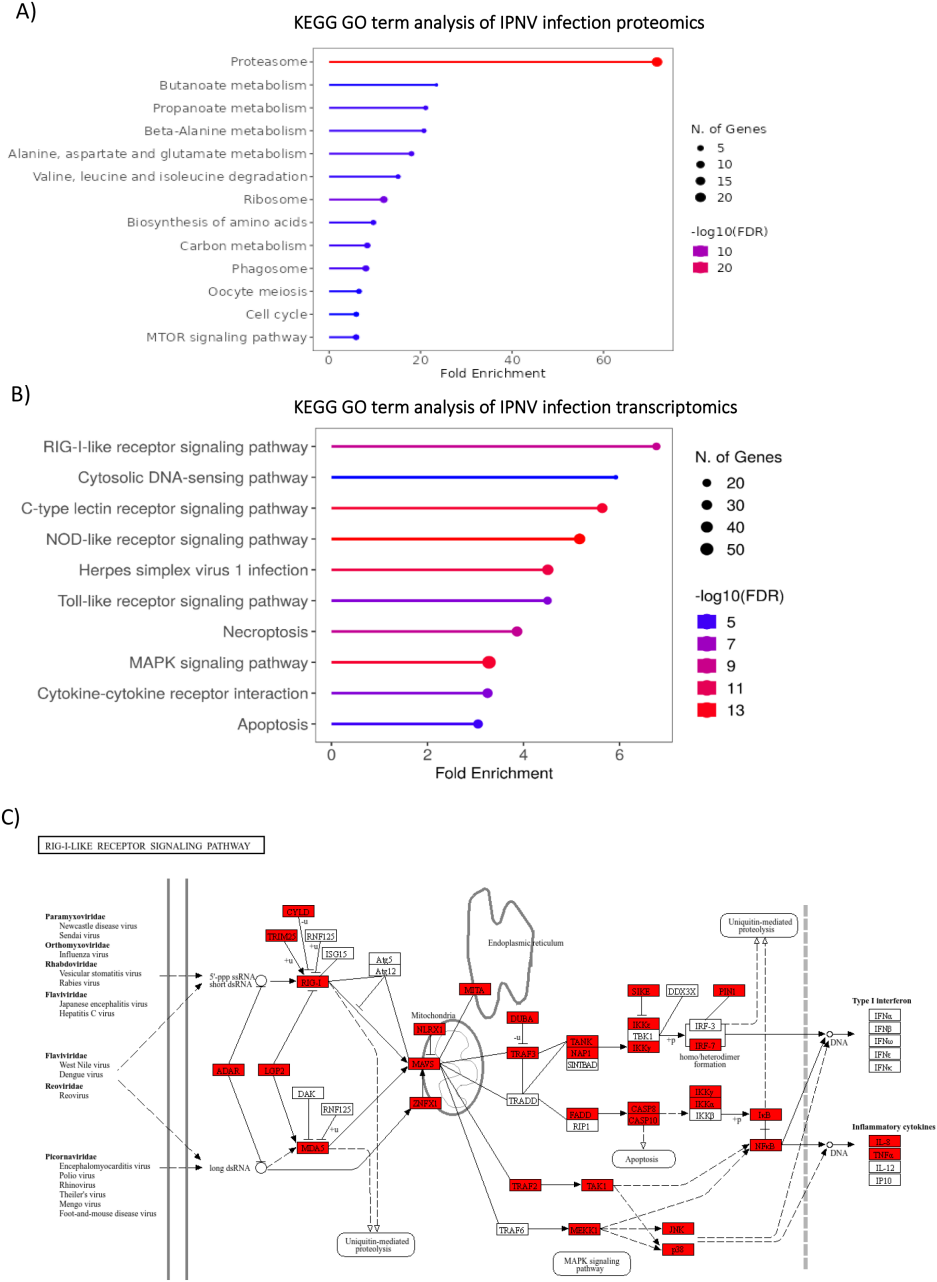
GO and KEGG analysis of IPNV infection**(A)** GO Term enrichment analysis of IPNV infection, 48 hours post infection **(B)** Enriched GO term analysis of significantly upregulated transcripts (padj value <0.05, FC >2) from IPNV infection at 48 hours post infection. GO Term analysis was performed on closest homologs of salmon genes in model species; Zebrafish. **(C)** KEGG pathway annotation of zebrafish genes homologous to Atlantic Salmon genes upregulated in the RIG-LIKE receptor pathway in response to IPNV Infection. Upregulated genes are highlighted in red. GO term analysis and KEGG pathway annotation was performed with R shiny GO (66).

GO enrichment analysis of IPNV-infected cell proteomics (**Figure 6A**) reflects a strong response to the virus, with protein/amino acid metabolism and the proteasome being the main enriched pathways. This suggests a proteome reprogramming, typically of the formation of an immunoproteasome in response to infection, which is regulated by the ubiquitin pathway. Proteasome-associated genes were enriched >60 fold, in addition to downstream metabolic degradation processes of amino acids (e.g. Beta alanine metabolism, >20 fold enrichment), and amino acid functional group metabolism (e.g. Butanoate and Propanoate metabolism, >20 fold enrichment). Whilst the most enriched terms are degradation and metabolism pathways, some biosynthesis pathways – ribosome and amino acid biosynthesis – are also enriched.

GO term analysis of IPNV infected cell trasncriptomics (**Figure 6B**) suggests a strong reliance on the Pathogen-associated molecular pattern (PAMP)/Pattern recognition receptor (PRR) signalling pathways for virus detection, with several classes of PPR including RLR, CTLR, NLR, TLR and cytosolic DNA sensing pathways highly significantly enriched. The PAMP/PRR signalling pathway via the RIG-I-like receptor signalling pathway is upregulated in its entirety for IPNV infection, reflecting a strong transcriptomics response to this virus at these timepoints (**Figure 6C**).

### Ubiquitin-mediated transcriptional reprogramming in response to ISAV and IPNV infections

Weighted correlation network analysis (WGCNA) of ubiquitin-enriched proteomics and transcriptomics was utilised to identify clusters of genes putatively regulated by the ubiquitination of the proteins identified in our analyses. WGCNA was performed on a matrix combining the normalised expression and protein levels obtained in the RNA sequencing and proteomics datasets created in this study. The expression of the viral genes was included to distinguish real protein-gene associations of those driven by the level of infection.

**Figure 7:**
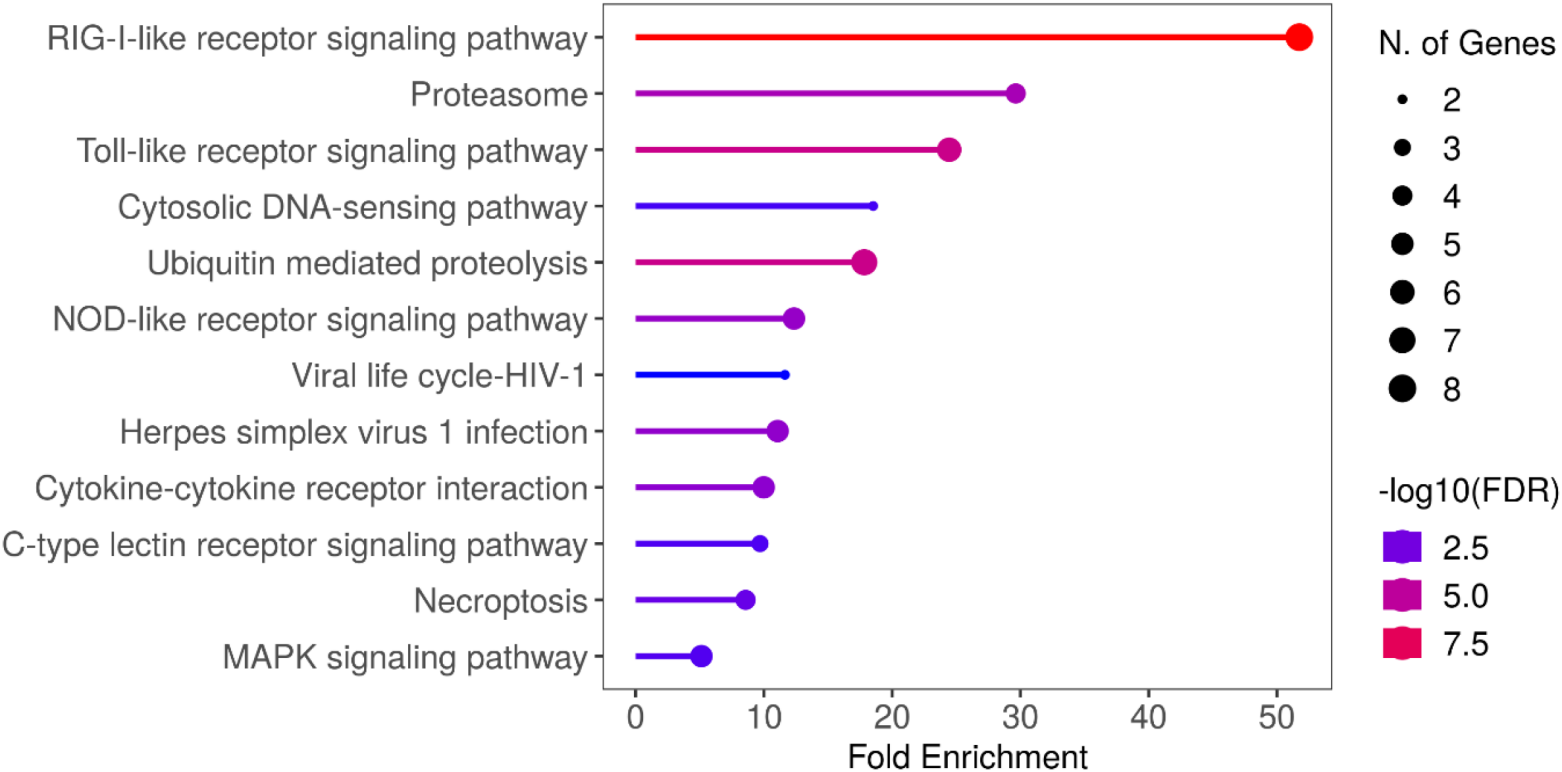
GO term analysis of trim25 containing node of WGCNA analysis. All infections and timepoints were pooled and WGCNA analysis was performed on RNA sequencing and proteomics analysis. Genes from node containing TRIM proteins (x7) was subjected to GO term analysis using Shiny Go 0.80. Zebrafish homologs of Atlantic Salmon genes were used for GO term analysis.

*Trim25* (both chromosome 2 and chromosome 12) genes were associated with a cluster containing 5 other TRIM-like genes, namely *trim33*, *trim37*, *trim69*, *trim71*, and *trim105*. RNA sensors RIG-I (dhx58) and MDA5 (ifih1), which are known viral double and single-stranded RNA sensors, respectively, and IFN pathway activators were also associated with this cluster, suggesting a potential activation pathway mechanism. Additionally, ubiquitin conjugating enzyme E2N (*ube2n*), a known interactor with TRIM25 in human, is present in this cluster suggesting a putative conserved E2/E3 ligase pair. A well-known antiviral interferon stimulated gene, Protein Kinase R (PKR, *eif2ak2*), is also associated with this cluster along with neddylation genes Nedd4 binding protein 1 (*n4bp1*) and Nedd4 like E3 protein ligase (*nedd4l*). The proteins associated with this cluster of genes include E2 ubiquitin-conjugating enzyme (B5X3E0), proteasomal subunit beta (A0A1S3LZZ6) and a probable ubiquitin ligase (A0A1S3L7A4). GO term analysis of this cluster was performed with R Shiny, revealing significant enrichment of RIG-Like, Toll-like and NOD-like receptor signalling pathways, in addition to multiple innate immune, cell cycle and viral infection pathways (**Figure 7**). The differentially ubiquitinated E2 ubiquitin-conjugating enzyme and probable ubiquitin ligase represent potential regulators of the antiviral immune response in Atlantic salmon.

Poly ubiquitin is differentially abundant upon infection with both ISAV and IPNV. The network analysis reveals ubiquitin protein (B5DGL7) is associated with multiple other components of post-translational regulation, including many post-translational modification pathway proteins; TRIM containing protein 47 (A0A1S3KR00), E3 ubiquitin ligase hectd1 (A0A1S3RC95), Nedd4 family interacting protein (B9EMY3), or Polyubiquitin/ISG15 like protein (Q29W12). Additionally associated with this node are multiple components of the proteasome - proteasome regulatory subunit 13 (B5DGU8), proteasome subunit beta (A0A1S3RIW7), Ubiquilin-4- like protein (A0A1S3NPQ8). Genes associated with this node also include ubiquitin ligases *trim31*, *trim32*, *trim36* and *trim55b.* This network suggests a coordinated regulation in the reprogramming of the post-translational modification/ubiquitination machinery in response to viral infection.

## Discussion

In this study ubiquitin-enrichment proteomics was merged with RNA sequencing to characterise the post-translational regulation of the cellular response of SHK-1 cells to infection with ISAV and IPNV. ISAV and IPNV are single-stranded and double-stranded RNA viruses, respectively, both of which pose significant economic threat to salmonid aquaculture. Emerging evidence highlights the importance of post-translational regulation in the host immune response to these viruses. Given the distinct nature, replication mechanisms, and infection kinetics of ISAV and IPNV, they were selected for this study based on their economic importance and previous evidence linking resistance to genes involved in post-translational modifications (14, 18, 21, 32, 34). However, due to these fundamental biological differences, any direct comparison of the host responses to the two viruses should be approached with caution.

A transcriptional response to infection for both viruses has been reported both *in vitro (* 14, 35) and *in vivo (* 17, 18, 36–39) in salmonids, however, investigation of post-translational modifications of proteins is limited despite evidence for its importance in determining the outcome of infection, particularly well-established in IPNV where Neddylation pathways are associated with resistance (21). By combining techniques, we have gained unique insight into the virus-induced changes to the post-translational landscape of the host, whilst also measuring the impact of the changing proteome on the host transcriptome.

Principle component analysis of SHK-1 transcriptomics in response to ISAV infection reveals a moderate response to ISAV, especially at 24 hours post-infection, whilst ubiquitin-enrichment proteomics shows severe changes in the host immune-proteome. Conversely, IPNV infection induces a strong transcriptional response to infection, with a concurrent reduction in the abundance of ubiquitinated proteins. Intriguingly, Control (uninfected) 24 hour and 48 hour samples cluster separately in transcriptome according to PCA analysis (**Figure 2B**). Possible explanations for this might be the differences between the routine culture conditions of SHK-1 cells, and the conditions required for viral replication. Typically, SHK-1 cells are grown at 20°C, with 10-20% foetal bovine serum, whilst ISAV replication is severely impeded at 20°C (40) and with high serum content, therefore reduced serum (2%) and lower incubation temperatures (15°C) are required for infection. The change in temperature and serum content of the cell culture media may explain the differences in the transcriptome of SHK-1 control cells between 24 hours and 48 hours, reflecting the metabolic adaptation of these cells to a new temperature and cell culture media. This is supported by the high degree of variations seen in heat shock proteins between timepoints.

### Infectious salmon anaemia virus infection induces a strong post-translational response before transcriptomic response

The cellular response of SHK-1 cells to ISAV shows a stronger post-translational basis than transcriptional response. The replication time of ISAV is undefined, however the muted transcriptional response observed at 24 hours followed by a greater response at 48 hours is consistent with the 24 hour lag phase proposed by Falk et al. (40), and Gervais et al (32) where ISAV infected SHK-1 cells were found to cluster with control group samples at 24 hours (32). It is also worth considering that the lag time in response to ISAV may be due to viral antagonism of the immune response. It has previously been suggested that the lack of strong interferon response for ISAV is evidence of a viral evasion mechanism (34), consistent with that seen in influenza; which belongs to the same Orthomyxoviridae family as ISAV (41). The question that follows is whether the lack of transcriptional response observed at 24 hours is due to a heavy reliance on post-translational activation of the immune response, or is symptomatic of viral antagonism to repress host antiviral response, for which mechanisms have been described previously for ISAV (42).

To the best of our knowledge, data presented here is the first evidence that during early infection the cell undergoes a post-translational response, which could drive the larger transcriptomic response observed at 48 hours. ISAV infection induced a rapid increase in total ubiquitinated proteins when analysed by western blot at the earliest timepoint of 24 hours, whilst transcriptional response was limited. This suggests that ubiquitination may be important in activating/regulating the initial host response to infection. Multiple components of the protein production and processing pathways, including the ribosome, endoplasmic reticulum and ubiquitination pathway showed increased ubiquitination at 24 hours post-infection. This suggests that rather than changing in abundance these proteins are undergoing significant changes in their ubiquitination state, indicative of a post translational modification-controlled response to infection. Whilst clear differences in the ubiquitination state of these proteins is shown, ubiquitin-enrichment proteomics picks up all ubiquitin-modifications, and therefore the change in ubiquitination state could reflect activation, as different ubiquitin linkage types can lead to different impacts on the substrate. Herein lies one of the limitations of the technique used, and further refinements would include the use of more selective ubiquitin linkage enrichment methods to determine the nature of the changing ubiquitome.

Differentially abundant ubiquitinated proteins of note upon ISAV infection include polyubiquitin, Polyubiquitin/ISG15 like protein and TRIM25-like protein. Polyubiquitin has previously been demonstrated to be upregulated *in vivo* in response to early infection (18), suggesting that the ubiquitination system is amongst the first to react to infection through its known activation of PRRs in fish (23). In addition to its immune regulatory role, ubiquitin is a master regulator of protein turnover, DNA repair and cell death (43). An increase in ubiquitin could therefore be indicative of the cytopathic effect of the virus, however cytopathic effect does not typically occur in SHK-1 cells infected with ISAV until 9 days post infection (44).

ISG15 is a viral PAMP inducible ubiquitin homolog (40% identical, 64% conservative substitutions (45)), as previously demonstrated by its induction by the dsRNA mimic, poly I:C (46). ISG15 has been demonstrated to regulate ubiquitination by competing for proteasome targeting ubiquitin sites in substrate proteins (47), and increasing evidence demonstrates the capability of ISG15 conjugation to targets substrates for degradation (48). The role of ISG15 is not well understood, but ISG15 has been suggested to perform a multitude of roles in homeostasis, including immune regulation, and is known to be transcriptionally upregulated by ISAV infection in Atlantic salmon (14). It remains to be established whether the ISG15 protein can be ubiquitinated, hence its presence in the data presented here; or whether the Ubiquilin enrichment utilised in this study can also enrich for ISG15 due to its similarity to ubiquitin. What is clear is that Polyubiquitin/ISG15 like protein is rapidly increasing in abundance post-infection, yet whether this is an anti-viral response or a pro-viral antagonism (via competing for ubiquitin-binding sites) of the immune response remains unclear.

UBR4 is an E3 ubiquitin ligase involved in protein quality control pathways in the cytoplasm. Despite mainly functioning in this quality control role, it has been described by Morrison et al. that co-opting of UBR4 by Dengue Virus (DENV) into antagonising type 1 IFN antiviral response by degrading STAT2, therefore antagonising interferon response – as such the presence of UBR4 is crucial for DENV replication (49). In contrast, UBR4 has also been shown to induce K48-linked polyubiquitination and degradation of ORF9b of SARS-CoV-2, thereby attenuating ORF9b antagonism of the IFN immune response (29). In our proteomics data UBR4 increases in abundance upon ISAV infection, whilst decreasing in abundance upon IPNV infection. Concurrently, there are no detected changes in UBR4 gene expression. This suggests that the change in abundance due to infection is most likely a change in ubiquitination state of UBR4, potentially allowing ISAV, a virus with demonstrated antagonistic non-structural proteins, to escape interferon response. Taken together, the proteomics data suggest that UBR4 has a role in viral antagonism via manipulation of its ubiquitination state, although whether it is acting in a pro-viral manor or anti-viral manor is unclear.

RNF31 is a component of the linear ubiquitin chain assembly complex (LUBAC) (50). LUBAC acts as a negative regulator of RIG-I RNA sensing of RNA viruses (26) by degrading TRIM25, thereby suppressing the IFN induction pathway. RNF31-like protein increases in abundance upon ISAV infection, whilst decreasing in abundance upon IPNV infection. An increase in abundance may be indicative of suppression of the RIG-I signalling pathway by ISA virus (typically by K48-mediated proteasomal degradation), or it may also be indicative of ubiquitin-based activation of LUBAC (typically by K63-linked ubiquitin activation). Further work should determine the type of ubiquitination that LUBAC undergoes upon viral infection to determine whether the change in abundance is host-induced degradation of LUBAC, enabling RIG-I signalling, or virus-induced activation of LUBAC.

In addition to an increase in ubiquitinated proteins associated with protein production and processing pathways, GO term enrichment analysis of upregulated genes highlighted multiple pattern recognition receptor pathways upon ISAV infection. Both extracellular receptors (CLR and TLR’s) and cytosolic receptors (NLR and RLR) genes are significantly enriched, suggesting detection of viral pathogen-associated molecular patterns (PAMPs) in the cytosol of the cell by NLR and RLR pathways, in addition to endosomal/extracellular sensing by TLR and CLRs.

### Conserved and fish-specific TRIM proteins play a major role in response to ISAV in Atlantic salmon

TRIM25 is a RING E3 ligase belonging to the TRIM gene family, defined by their conserved Ring domain, B-box domain and coiled coil domain (RBCC). TRIM proteins catalyse ubiquitination, ISGylation and sumoylation of a broad range of substrates in a diverse range of cellular processes (51). TRIM25 is a known regulator of innate antiviral immune response in model species, though its mechanism remains controversial in model species and unknown in salmonids (25, 52). TRIM25 genes have repeatedly been demonstrated to be induced by ISAV infection *in vitro (* 14, 32) and *in vivo (* 18, 34) at the transcriptomic level, however, our data represent the first evidence of regulation of TRIM25-like at the proteomic level in fish. Taken together with current literature, transcriptional and proteomic data provide clear evidence of the virus inducibility of Atlantic Salmon TRIM25 (*ssTRIM25*). However, our data suggest a preferential induction of the chromosome 2 copy of *ssTRIM25*. For both ISAV and IPNV infections ssa02 *ssTRIM25* is upregulated more than ssa12 *ssTRIM25* (fold change and significance) at 48 hours post infection. This expression pattern is similar to that observed in Clark et al. (53), where only the chromosome 2 copy of trim25 (ENSSSAG00000054152) is differentially expressed in response to infection both *in vitro* and *in vivo* when stimulated with poly I:C. Taken together, this suggests some degree of either subfunctionalisation or neofunctionalisation, as previously described by Lien et al (33). Further work should validate whether only the chromosome 2 copy is functional or if there is a different ohnolog expression pattern in different cell and tissue types, or in response to different stimuli.

Many TRIM proteins are significantly upregulated in response to ISAV infection. *Boudinot et al. (* 54) characterised the TRIM genes in teleost fish into trim25-like genes, trim16-like genes, novel Fish trim (ftr) and ‘bloodthirsty-like’ TRIMs (btrs), of which at least one of each type is upregulated in response to ISAV infection. This confirms the importance of all four trim gene types for functional immune response in fish (51). TRIM genes have been well-characterised in model systems, where they have been demonstrated to have direct antiviral roles – e.g. TRIM21 binds to virus-bound antibodies (31, 55). They can also initiate the establishment of an antiviral state by regulating cell signalling and can even have immune antagonistic roles (56). It is clear then that the TRIM gene family that expanded rapidly at the evolution of the modern innate and adaptive immune system (56) plays an important role in the activation and regulation of the salmon immune response to viruses. Further work should characterise and confirm the inducibility and the functions of these virus-induced E3 ubiquitin ligases to identify targets for disease-resistant gene-edited fish, and virus-propagation cell lines for vaccine production.

### Infectious pancreatic necrosis virus infection induces a strong transcriptional response followed by proteasomal reprogramming

Characterisation of the post-translational response to IPNV infection shows a mild deubiquitination of host proteins at both 24 and 48 hours post-infection. Several protein components of the Neddylation pathway decreased in ubiquitination abundance post-infection, specifically Nedd4 family interacting protein 1 (Ndfip1), which is a regulator of HECT E3 ubiquitin protein ligases including Nedd4 (57) and a negative regulator of RIG-I-dependent immune signalling by degrading MAVS (58). Therefore, the reduction of abundance of Ndfip1 could be associated with the upregulated transcription of the RIG-I pathway. Nedd8 is also reduced in abundance upon infection with IPNV. Nedd8 has been previously associated with genetic resistance to IPNV in Atlantic salmon, where knock-out of NEDD-8 activating enzyme (*nae1*) induced a significant reduction in IPNV replication (21). In combination with our results this highlights the important role of the neddylation post-translational response to IPNV infection. The data suggests that the reduction in ubiquitination state and/or abundance of ubiquitinated Nedd8 may be indicative of increased stability of Nedd8 due to reduced proteasomal targeting via the UPS pathway.

Both ISAV and IPNV infections induce an increase in the abundance of an associated Polyubiquitin/ISG15 like protein (Q29W12), which correlates with the increased transcription of the relevant *ISG15* genes. ISAV 48 hours post infection and IPNV 24 and 48 hours post infection all have a significantly upregulated Polyubiquitin/ISG15 like protein (Q29W12). ISG15 has been proposed as a negative regulator of ubiquitination by competing for substrate binding sites (59) – this may in-part explain why Polyubiquitin/ISG15 like protein is much more abundant whilst the ubiquitination state of many proteins is reduced upon IPNV infection. In addition to a possible antagonist role with ubiquitin, free ISG15 has been demonstrated to act as a negative regulator of Nedd4 in humans (60) – this may explain the large increase of ISG15 at 48 hours post infection, paralleled with the large reduction in abundance of Nedd4 family proteins. What remains clear is that post-translational modification by ubiquitin and ubiquitin-like homologs – neddylation, ISGylation and SUMOylation – are important regulators of early antiviral interferon-mediated immune response, and their regulation may temporally occur before a transcriptional response to pathogen invasion.

### Both infectious salmon anaemia virus and infectious pancreatic necrosis virus trigger activation of the RLR pathway

Both Infectious Salmon Anaemia Virus and Infectious Pancreatic necrosis virus induce significant GO term enrichment of RIG-Like receptor pathways. The RIG-I-like pathway is initiated by a family of cytosolic RNA helicases which act as pattern recognition receptors (PRR). These PRRs are activated upon detection of single-stranded RNA containing a terminal 5’ triphosphate group and/or dsRNA pathogen-associated molecular patterns, leading to a signalling cascade which results in production of IFN-I and the establishment of an antiviral state. The detection of these PAMPs from RNA viruses is mediated by either RIG-I or MDA5 in teleost fish, both of which have also been demonstrated to be conserved in Atlantic salmon (61).

Both ISAV and IPNV infection induced practically all the genes in the annotated RIG-I pathway, yet the receptors for viral PAMPS differed between infections. ISAV infection resulted in upregulation of RIG-I exclusively, whilst IPNV infection-induced upregulation of both RIG-I and MDA5. It is established that RIG-I preferentially recognises single-stranded RNA via the 5’ phosphate cap, in addition to short double-stranded RNA (62), whilst MDA5 seems to preferentially bind long dsRNA (63). This is consistent with the literature regarding MDA5 sensing of double-stranded RNA viruses, such as IPNV, whilst ISAV is a single-stranded RNA virus and as such does not lead to MDA5 activation. However, it is likely that both dsRNA viruses and ssRNA viruses exist at various stages in the replication cycle as single and double stranded RNA, and as such it is to be expected that both RIG-I and MDA5 pathways may be redundant and may be activated upon a broad range of infections.

## Conclusions

Post-translational modification of proteins by the addition of ubiquitin (and ubiquitin-like proteins such as ISG15) has an important role in the initiation and regulation of early antiviral immune response in Atlantic salmon in response to both IPNV and ISAV. For ISAV infection, ubiquitination response precedes a strong transcriptomic response to infection. In contrast, upon IPNV infection there is a strong transcriptional response, concurrent with a decrease in the majority of ubiquitinated proteins. Ubiquitination of several proteins could be associated with the regulation of RIG-Like, Toll-like and NOD-like receptor signalling pathways, linking post-translational modification state with the regulation of the antiviral response in this species. Ubiquitomics remains an understudied field in non-mammalian species, yet our results strongly suggest a key role of ubiquitin and ISG15 in the immune response of fish to viral infection. Further investigation of the role of this highly conserved molecule and validation of observations described herein *in vivo* is therefore needed to provide further insights into fish immunology.

## Materials and methods

### Cell culture and viral Infection

SHK-1 cells (ATCC 97111106) were propagated in L15 (Sigma-Aldrich, MA, USA) media supplemented with 5% FBS (Gibco, MA, USA), 40 µM of β-mercaptoethanol (Gibco, MA, USA), 4mM of glutamine (Gibco, MA, USA), and Penicilin-Streptomicin antibiotics (Gibco, MA, USA). At 80% confluence, cells were passaged using 0.25% of trypsin/EDTA, pelleted and split 1:3. Fresh media was added in 2:1 ratio with conditioned media. Viral stocks of ISAV and IPNV were generated and quantified on SHK-1 cells utilising TCID50. ISAV 390/98 was isolated from the first ISAV salmonid outbreak in the west of Scotland in 1998, and was at passage 9 at time of infection. IPNV isolate V0512–1 was used at passage 2, originally isolated from naturally occurring IPNV infection of Atlantic salmon in Scotland in 2005.

Cells were counted using the Improved Neubauer haemocytometer (Sigma-Aldrich, MA, USA) and seeded overnight at 20oC in 6 well plates. To inoculate cells media was replaced with L15 media with 2% FBS containing virus for 3 hours at 15 °C, cells were washed and media was replaced with 2% FBS L15 and incubated at 15 °C for either 24 or 48 hours. Infections were performed at MOI 0.1 for ISA virus and 0.01 for IPN virus; a control plate without virus was also made. Cells were collected at 24 and 48 hours post-infection, using trypsin-EDTA and washed once with PBS before storing the cell pellet at -80 °C either with or without TRIzol^®^ reagent (Invitrogen™). Total RNA from samples kept in TRIzol^®^ were extracted using Direct-zol™ RNA Microprep (Zymo research, Irvine, USA) with DNase I treatment before storing at -80 °C for transcriptomic analysis. The RNA quality of each sample was checked using 4200 Tape station (Agilent) and Nanodrop, and only samples with RNA Integrity number (RIN) > 7 were used. 4 biological repeats were analysed for each infection, at each timepoint for RNA sequencing and proteomics.

### Western blot

Cells were harvested at 24 and 48 hours post infection. Infected and non-infected SHK-1 cells were lysed in extraction buffer 1X PBS with 0.05% Igepal CA-630 (formerly NP-40), 0.05% Triton, 10 mM NEM, 50 μg/ml TPCK, 50 μg/ml TLCK, 0.6 mM PMSF, 100 μM MG132, 200 μM Phosphate inhibitors cocktail 1 (Sigma). Homogenates were centrifuged at 8,500g at 4 °C for 15 mins to remove cellular debris. Samples were normalised for total protein content via BCA assay then subjected to electrophoresis on 8% Tris-Glycine SDS gel. Protein bands were transferred onto nitrocellulose membranes overnight at 2-8 °C and blocked with 5% Non-fat dried milk (NFDM). Ubiquitinated proteins were detected by immunoblotting with anti-ubiquitin antibody (mouse anti-Ubiquitin mAb, clone P4D1, 1:4000, 1% NFDM) and visualised with Anti-Mouse IgG HRP (Cell Signalling #7076, 1:3000, 1% NFDM). Anti-pan Actin (Cell signalling #4968, 1:1000, 1% NFDM), was used as housekeeping loading control. Total protein loading was confirmed with Ponceau staining (**Supplementary Figures 1A, B**).

LICOR image studio software was used to quantify western blot bands. Ubiquitin bands were normalised against actin housekeeping control. Groups were compared using One-way ANOVA. Where significant differences were found (p value <0.05), multiple comparisons were performed using a Dunnets multiple comparison test.

### Mass spectrometry of ubiquitinated proteins

Samples for proteomics were analysed by the Roslin Proteomics and Metabolomics facility. Sample were subject to purification using HaloTag^®^ magnetic beads-bound Halo-ubiquitin (Genbank NM_053067.2, MRC PPU Reagents and Services, School of Life Sciences, University of Dundee) following the protocol described by Emmerich et al. (64) prior to mass spec analysis. Samples were prepared for bottom-up analysis by reduction of disulphide bonds with TCEP and cysteine residues alkylated to prevent reformation with chloroacetamide. Samples were acidified with phosphoric acid and digested into peptides with trypsin via S-Trap digestion, and were analysed by LC-MS/MS using tims TOF in a data dependent acquisition. Raw mass spectral data was processed using PEAKS Studio X-Pro Software (Bioinformatics LTD). Search was performed against Uniprot Atlantic Salmon sequence database containing 47,722 entries. Data of proteins detected in all samples were processed using Perseus software (65). Gene ontology (GO term) analysis was performed on mass spectroscopy data with R ShinyGO (66). Proteomics raw data and results are available in **Supplementary File 1**.

### RNA sequencing

Total RNA from experimental material was used to construct polyA-enriched RNA libraries using Illumina’s TruSeq RNA Library Prep kit v2, and libraries were sequenced on an Illumna Novaseq 6000 instrument as 150 bp paired-end reads. Library construction and sequencing was performed by Novogene. The raw reads were quality filtered using Fastp v.0.21.4 (67). Adapter sequences were removed, reads with less than 30 bp were discarded, and low-quality bases (Phred score < 15) were filtered out. The resulting reads were pseudo-aligned against the Atlantic salmon reference transcriptome (Ensembl Ssal_v3.1) using Kallisto v0.46.1 (68). Transcript-level expression was imported into R version 4.3.2 and summarised to the gene level using tximport v1.30.0. Differential expression analysis was performed using Deseq2 v1.42.0 (69), and genes with adjusted p-values < 0.05 were considered differentially expressed. Gene Ontology (GO) enrichment analyses were performed using the web tool ShinyGO v0.77 (66) for KEGG pathway annotations. Enriched GO terms analysis was performed on the closest human homolog to the Atlantic Salmon genes that were upregulated upon infection, due to a much higher degree of annotation of the zebrafish (*Danio rerio*) genome. RNA sequencing raw data has been uploaded to the NCBI Short Read Archive (SRA) database under BioProject accession number PRJNA1308608, and the differential expression results are available in **Supplementary File 2**.

### Network correlation analysis

A matrix containing normalised gene expression and protein abundance values for each sample was generated, and a weighted network correlation analysis was performed using the WGCNA package v1.72 (70, 71) in R version 4.3.2. Briefly, the matrix was transformed into a weighted correlation network using a power of 3, which was then clustered into modules of highly correlated genes and proteins, allowing a minimum of 30 genes/proteins per cluster. A functional enrichment analysis was performed on specific modules using ShinyGO version 0.77 (66).

## Data Availability

The RNA-seq data presented in the study are deposited in the Short Read Archive (SRA) repository of the NCBI, BioProject accession number PRJNA1308608. All other data is available within the **Supplementary Files**.
