# Supplementary material for: Characterisation of post-translational and transcriptional reprogramming of the immune response to ISAV and IPNV infections in salmon head kidney cells
Source: Front Immunol. 2025 Oct 17;16:1532917. doi: 10.3389/fimmu.2025.1532917 (PMC12575339; doi:10.3389/fimmu.2025.1532917)

Supplementary figure 1a- 24 hour western blot Ponceau stain. Wells included in main paper are annotated.


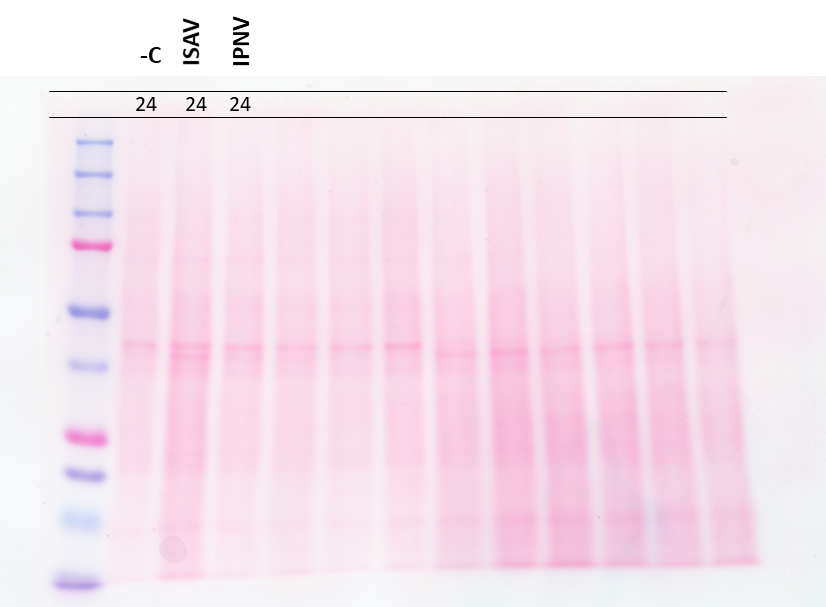


Supplementary figure 1b- 48 hour western blot Ponceau stain. Wells included in main paper are annotated.


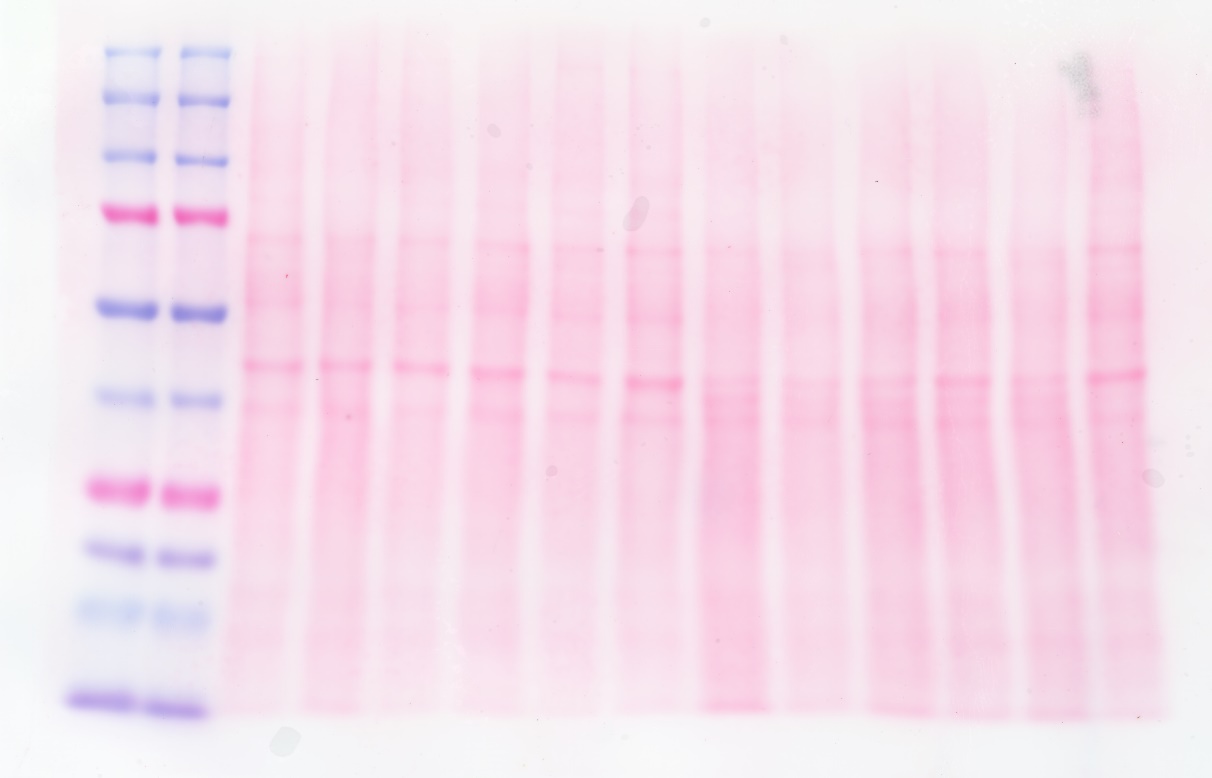


**-C**

**ISAV**

**IPNV**

48

48

48


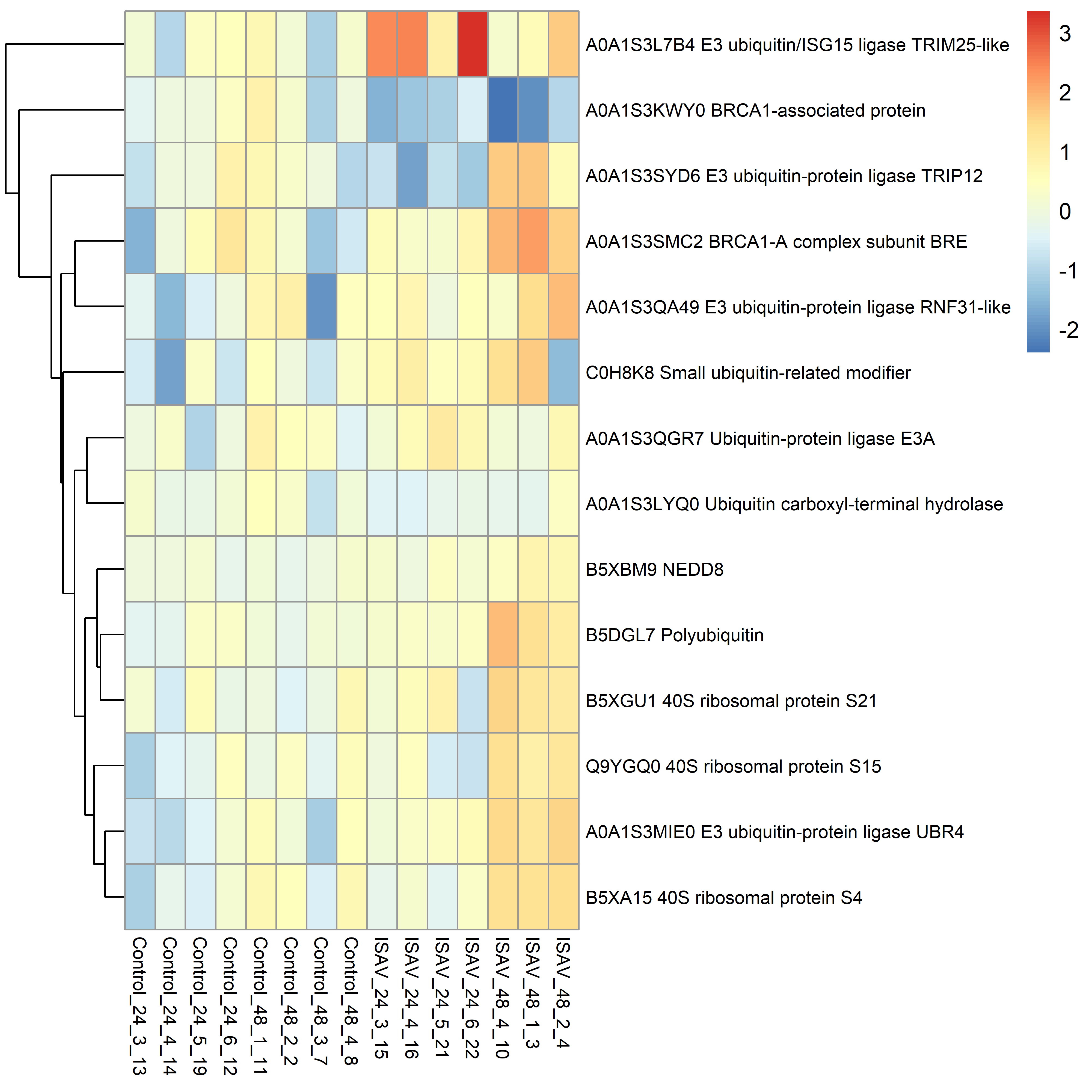
Supplementary figure 2 – ISAV (top) and IPNV (bottom) Heatmap of individual samples from proteomics. Colours represent log2FC. Samples are clustered by infection condition.


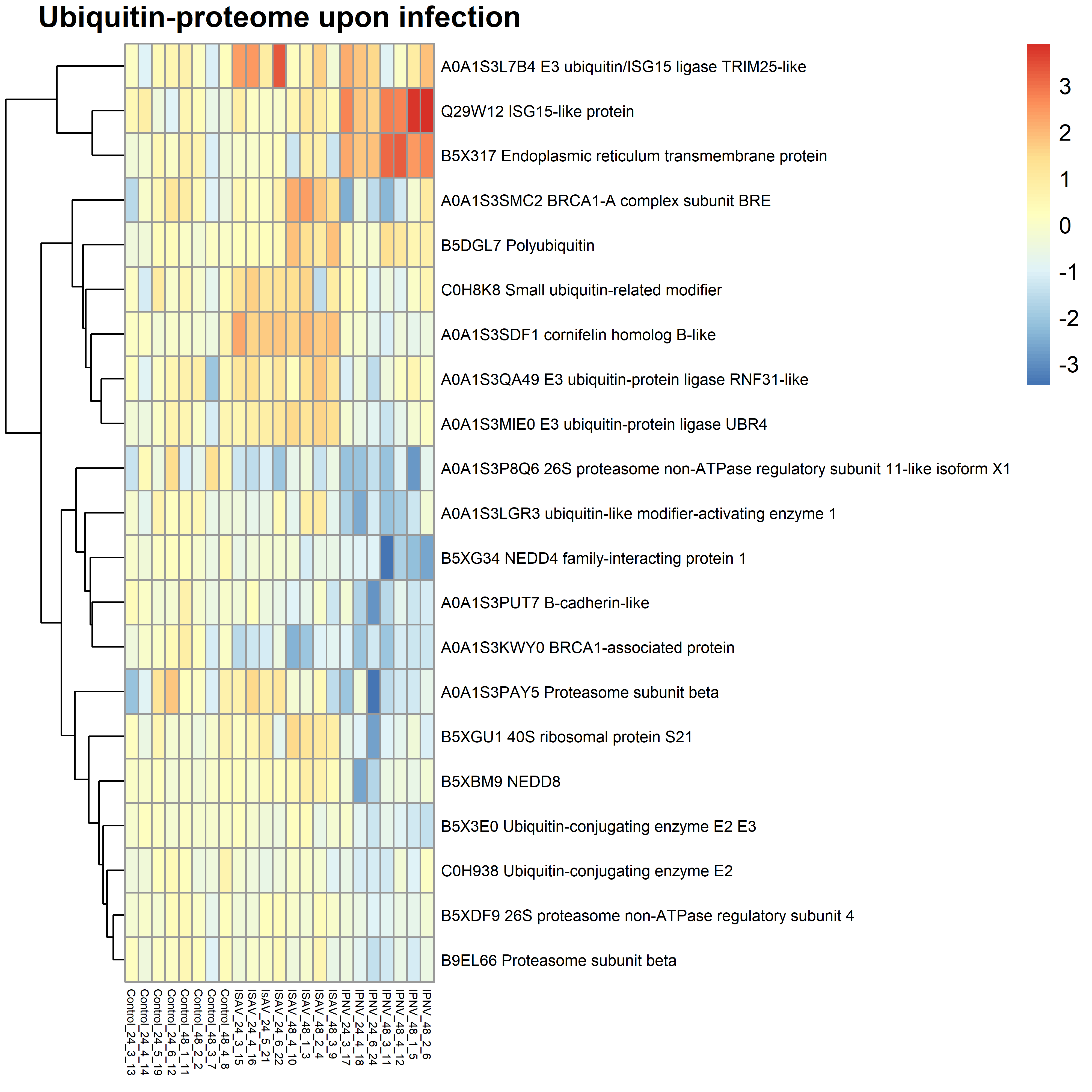


Supplementary figure 3 – Venn diagrams of differentially abundant proteins detected by MS proteomics during infection with ISAV and IPNV.


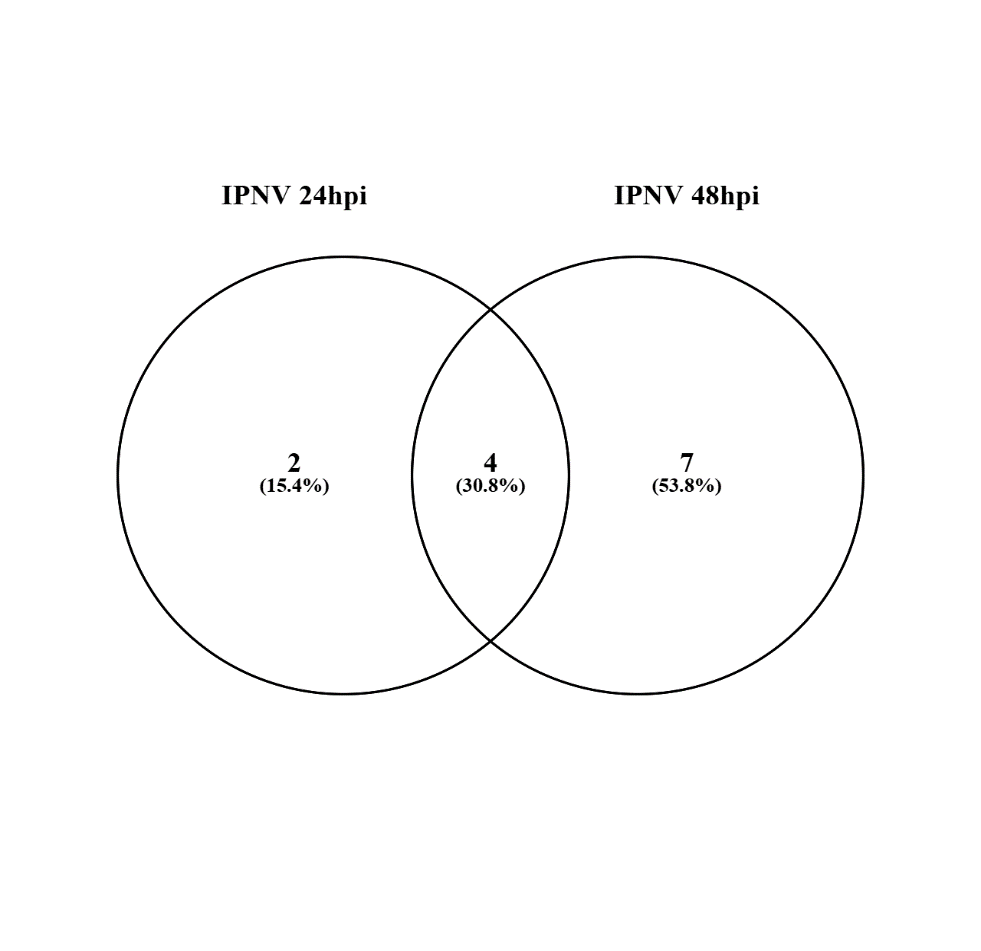

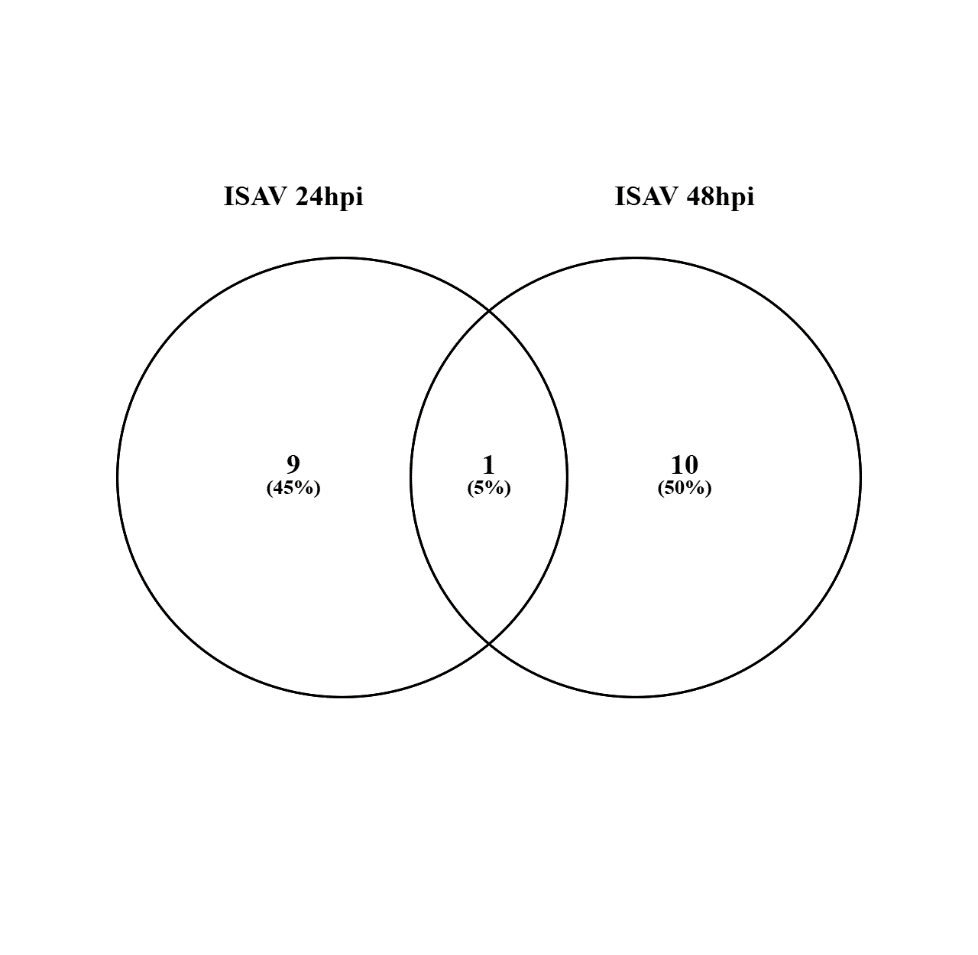


IPNV significantly increased abundance (FC >2)

IPNV significantly decreased abundance (FC >2)

ISAV significantly decreased abundance (FC >2)

ISAV significantly increased abundance (FC >2)


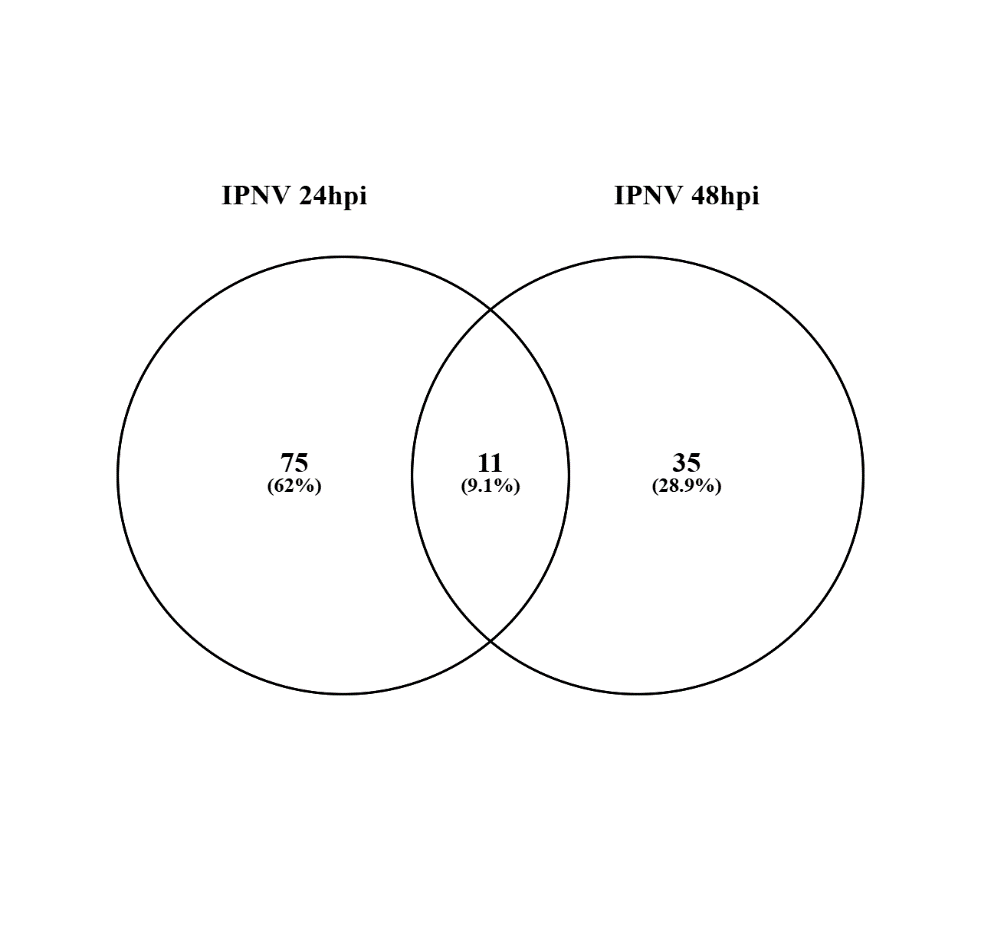

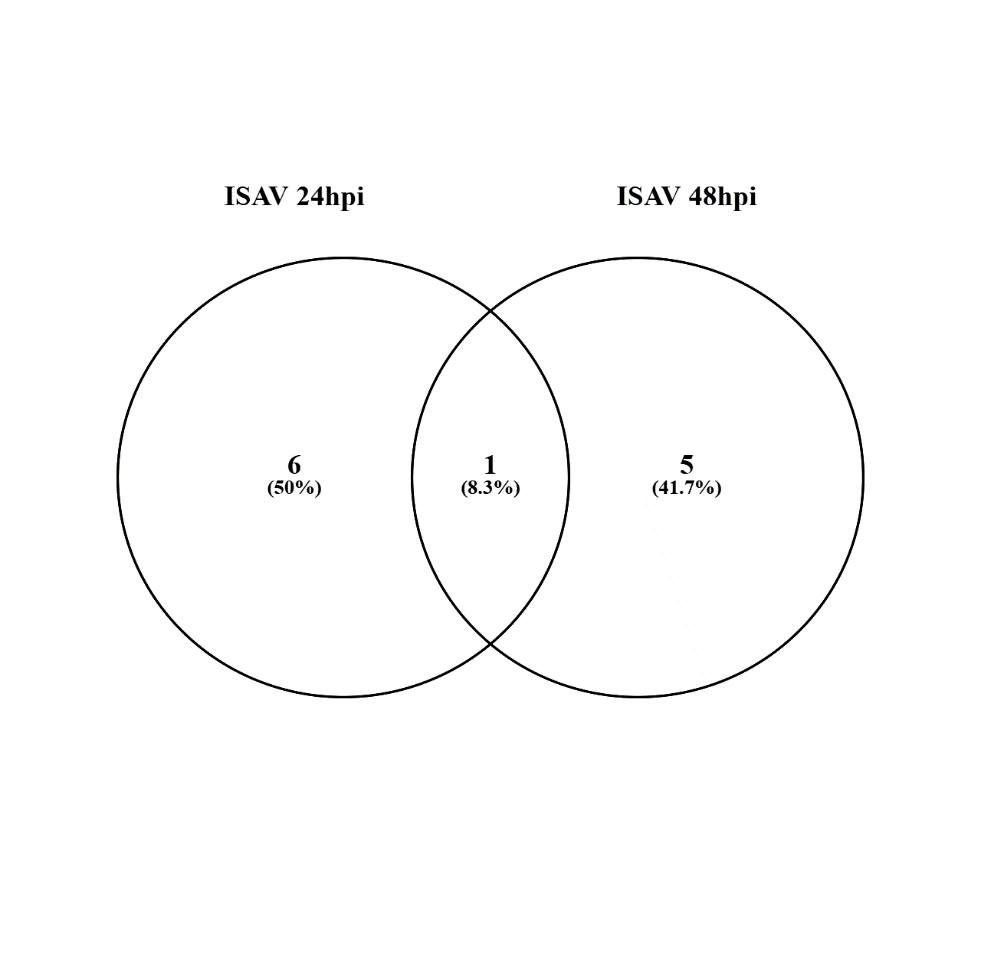

Supplement: Supplementary file 3 [file Table3.docx]
